# A potential prospect: The novel treatment of intrapleural saline irrigation with intrapleural tyloxapol in treating thoracic empyema

**DOI:** 10.1002/rcr2.70000

**Published:** 2024-08-10

**Authors:** Mas Fazlin Mohamad Jailaini, Noor Amirah Saini, Mohd Jazman Che Rahim, Mohamed Faisal Abdul Hamid

**Affiliations:** ^1^ Respiratory Unit, Faculty of Medicine Universiti Kebangsaan Malaysia (UKM) Kuala Lumpur Malaysia; ^2^ Respiratory Unit, Faculty of Medicine Universiti Sains Malaysia (USM) Kota Bharu Malaysia

**Keywords:** empyema thoracis, intrapleural saline irrigation, tacholiquin, tyloxapol

## Abstract

The treatment for empyema thoracis has been evolving over the years, including the usage of intrapleural fibrinolytic therapy (IPFT), for example, alteplase with intrapleural deoxyribonuclease (DNase) to enhance the drainage of pleural effusion. Here, we report two cases of thoracic empyema that were successfully treated with intrapleural saline irrigation and intrapleural tyloxapol apart from parenteral antibiotics as the pillar of the treatment. Both patients averted surgical procedure (decortication) and were discharged well. Upon follow‐up, both cases showed clinical cure, biochemical recovery, and radiological improvement, indicating a good treatment outcome. This is the first reported cases on combination of saline irrigation with tyloxapol as alternative treatment for pleural infection.

## Graphical abstract

Here, we report two cases of thoracic empyema that were successfully treated with intrapleural saline irrigation and intrapleural tyloxapol apart from parenteral antibiotics as the pillar of the treatment. Both patients averted surgical procedure (decortication) and were discharged well. Follow‐up also showed a good treatment outcome.

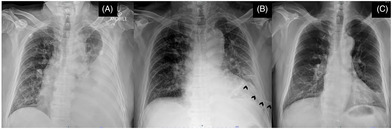

## INTRODUCTION

Thoracic empyema, a challenging pleural infection, leads to substantial health and economic burdens with prolonged hospitalizations and potential surgeries. Intrapleural tissue plasminogen activator (TPA) combined with dornase alfa (DNase) enhances fluid drainage, reducing surgical referrals and hospital stays, but their high cost and limited accessibility hinder widespread use.^1^, ^2^

Hooper et al. demonstrated that intrapleural saline irrigation improves pleural drainage and decreases surgical referrals as an alternative.^3^ Pleural irrigation has been incorporated into the management algorithm for pleural infections in cases where clinicians assess that patients are at high risk of bleeding from intrapleural fibrinolytics.^4^ However there was no studies on combination with intrapleural enzyme therapy.

Tyloxapol, a mucolytic synthetic surfactant primarily used for bronchopulmonary secretions.^5^ Unlike DNAse, the usage of tyloxapol in treating pleural infection via intrapleural route is not established. As of date, only literature by Provan did mention the usage of tyloxapol in treating pleural empyema.^6^ It was used intrapleurally with chloramphenicol for pleural empyema treatment.^6^ Nonetheless, the outcome could have been more successful. By adapting the concept and mechanism of action of tyloxapol, we report two culture‐positive pleural empyema cases treated with intrapleural tyloxapol (tacholiquin) combined with saline irrigation, following standard best‐practice management. This innovative approach aims to optimize treatment outcomes while considering cost‐effective strategies for managing thoracic empyema.

## CASE 1

**FIGURE 1:**
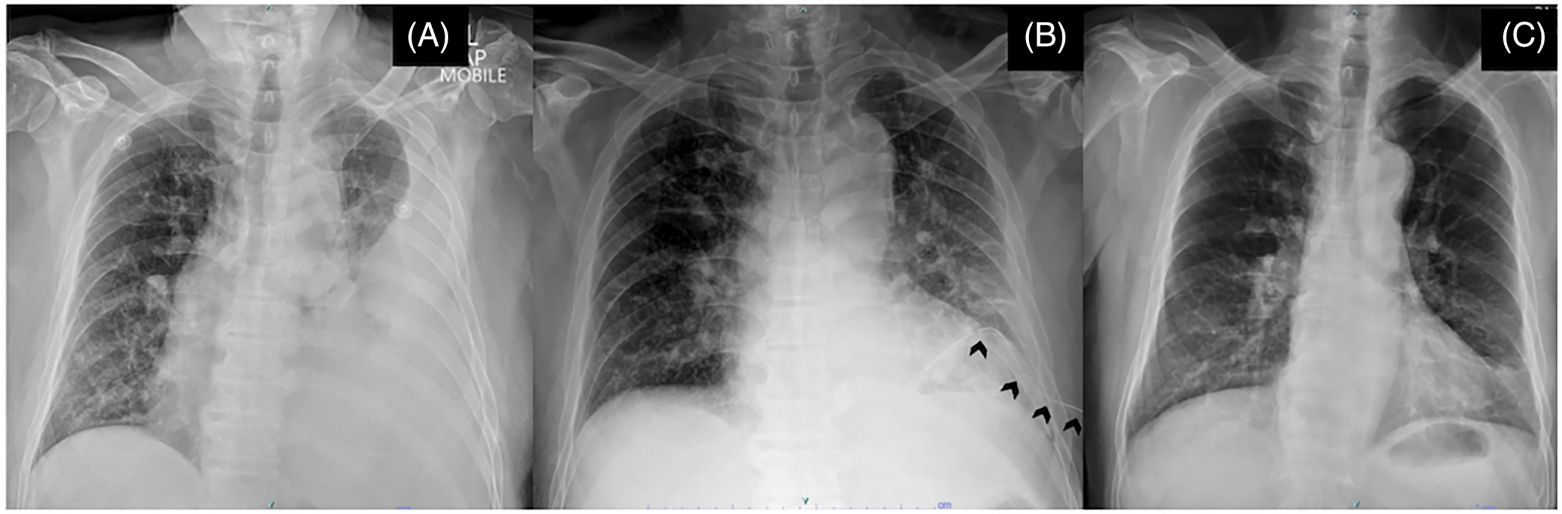
(A) Chest radiograph showed a moderate pleural effusion. (B) Chest radiograph after completion of three cycles of intrapleural saline irrigation and intrapleural tyloxapol showed significant effusion of left pleural effusion; the ICC (arrow) was in‐situ. (C) Chest radiograph on follow‐up 6 weeks later showed resolution of pleural effusion.

A 64‐year‐old man, an ex‐smoker, who is diabetic, presented to our emergency department with a 1‐week history of productive cough, fever and dyspnoea on exertion. On respiratory examination, there is reduced chest expansion with reduced breath sound at the left lung. His chest radiograph (Figure 1A), together with a complementary bedside ultrasound of the left hemithorax, confirms echogenic left pleural effusion with minimal septations within the pleural cavity. Subsequently, via ultrasound guidance, an intercostal chest catheter (ICC) size 12Fr was inserted by clinician and drained 1.3 L of pus.

Pleural fluid for lactate dehydrogenase (LDH) was 1710 U/L, and cytology showed an abundance of mixed inflammatory cells with no malignant cells seen. Pleural fluid culture grew *Streptococcus intermedius* and was sensitive to penicillin. Blood culture and sputum culture were negative. The initial white cell count (WCC) and C‐reactive protein (CRP) were 22.4 × 10^9^/L and 20.92 mg/dL, respectively. The drainage of pleural fluid was minimal despite regular flushing of the ICC.

After ensuring the patency of the ICC, the first cycle of intrapleural saline irrigation 250 millilitres was instilled within 1 h, and 45 min later, intrapleural tyloxapol (200 mg) diluted with 30 mLs saline was given over 5 min, which was then allowed to dwell for 45 min and then unclamped to allow free drainage till next cycle. He received three cycles of intrapleural saline irrigation and intrapleural tyloxapol (given 12 h apart), and the final total pleural fluid drained was 3.3 L with remarkable improvement of the chest radiograph (Figure 1B). The ICC was removed after 4 days. He received 2 weeks of intravenous amoxicillin/clavulanate acid followed by oral treatment for another 4 weeks with a significant reduction in his septic parameters as well (TWCC reduced to 7.5 × 10^9^/L and CRP 4.61 mg/dL. A follow‐up chest‐radiograph done 6 weeks later showed a resolution of pleural effusion (Figure 1C).

## CASE 2

**FIGURE 2:**
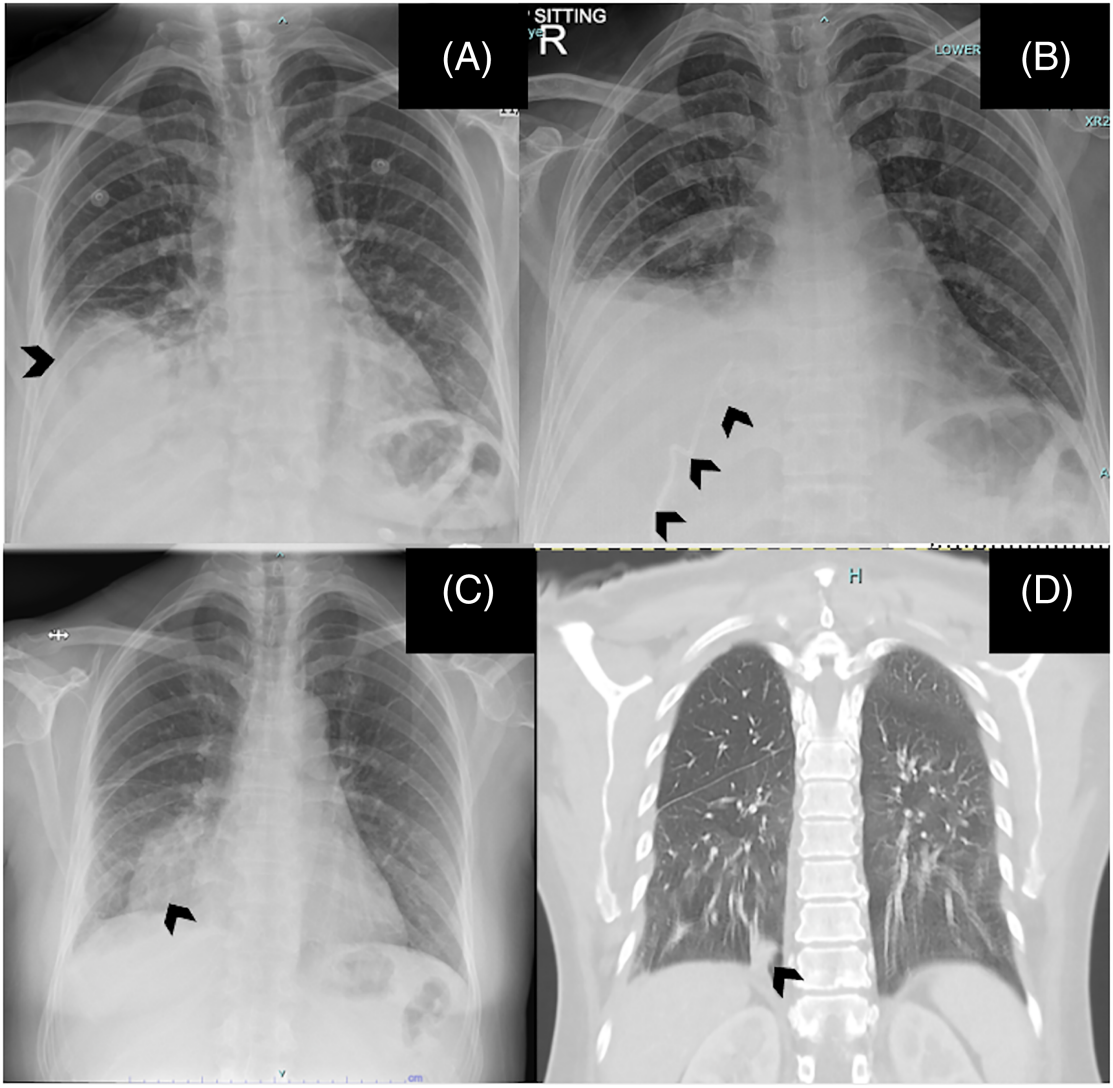
(A) Chest radiograph revealed right lower zone opacity with blunting of costophrenic angle. (B) Chest radiograph showed ICC in‐situ (arrow) with persistent pleural effusion. (C) Chest radiograph showed significant improvement of right pleural effusion after completion of intrapleural therapy; with presence of atelectatic lung (arrow). (D) CECT Thorax on follow‐up showed resolution of effusion with rounded atelectasis right lung (arrow).

A 47‐year‐old woman, who was an ex‐smoker without any co‐morbidities, presented to the emergency department with 3 days history of fever, cough and shortness of breath. On examination, she was febrile, with her vital signs within normal limits, and respiratory examination showed reduced breath sounds and dullness to percussion over the right lower zone. A chest radiograph (Figure 2A) showed right pleural effusion and a complementary bedside ultrasound thorax revealed hyperechoic effusion with multiple septations within. Anticipating a difficult insertion, an 8Fr‐size ICC was inserted by the interventional radiologist under image guidance into the right pleural space. This drained pus and allowed free flow, facilitated by regular chest drain flushing.

Pleural fluid analysis showed high LDH (4207 U/L) and low glucose (<0.3 mmol/L) and the cytology was negative for malignant cells. The pleural fluid culture was negative; however, her blood culture showed *Streptococcus pneumoniae*, which was sensitive to penicillin. Intravenous amoxicillin/clavulanate was given while in the ward as the standard best‐practice management for pleural empyema. The initial total WCC were 15.1 × 10^9^/L, and the CRP was 29.69 mg/dL. Unfortunately, the pleural fluid drainage was suboptimal over 1 week, with persistent loculations, and she remains febrile (Figure 2B). She received three cycles of intrapleural saline irrigation with intrapleural tyloxapol 12 h apart, and the total pleural fluid drained was 1060 mL. Following that, there was a remarkable radiological improvement (Figure 2C) coupled with a significant decline in the septic markers (total white cell counts of 4.9 × 10^9^/L and CRP reduced to 2.54 mg/dL). She was discharged after receiving 2 weeks of intravenous antibiotics followed by 4 weeks of oral antibiotics. Follow‐up in the outpatient clinic after 1 month also showed good clinical outcomes, and a contrasted enhanced Computed tomography (CECT) scan of the thorax showed resolution of pleural effusion with minimal rounded atelectasis at the right lower lobe (Figure 2D).

## DISCUSSION

Pleural irrigation trial (PIT) demonstrated an alternative treatment for pleural infection. In the trial, a physiological 0.9% sodium chloride was administered into the pleural cavity via a chest tube, a three‐way tap and a drip stand.^3^ The instillation of intrapleural saline was given via gravity without the usage of an infusion pump for over 1 h; then, the chest tube was allowed to drain freely. The whole procedure was given three times a day for a total of nine irrigations.^3^ There are also other case reports available on intrapleural saline irrigation that show promising clinical outcomes when given as an adjunct to the standard care of treatment or as an alternative to surgical intervention.^7^, ^8^, ^9^ This indicates that 0.9% sodium chloride might have some adhesiolysis effects in treating complex pleural infections.

Tyloxapol, on the other hand, has the properties of mucolytics and is able to cleave the triglyceride‐rich lipoproteins, liquefying the mucus and hence making it less viscous. By implementing the mechanism of action of tyloxapol, we decided to administer tyloxapol intrapleurally in pleural infection patients as an adjunct to the standard care of treatment. One ampoule of tyloxapol contains 5 mL of 1% tyloxapol (1 mL = 10 mg), and it is readily available in Malaysia at an affordable price. The dose of intrapleural tyloxapol given in our patients was 200 mg (four ampoules) each cycle as per description by Provan.^6^ Thus far, to our best knowledge, there is no other research or studies on the usage of combination of pleural irrigation and intrapleural tyloxapol; therefore, further exploration and analysis on it is urgently needed.

Based on various literature reviews available together with our clinical experience, we include the stepwise procedures on instillation of intrapleural saline irrigation in combination with intrapleural tyloxapol given at our centre:

1. Established diagnosis of complex pleural infections/empyema.
2. Exclude the presence of any bronchopleural fistula.
3. Insertion of chest drain via radiological guidance (small bore intercostal chest drain is advocated in the current management of pleural infection as per BTS 2023 recommendations.^4^
4. Regular flushing of chest tube with 20 mL 0.9% saline three times per day.^10^
5. Ensure chest tube patency before instillation.
6. Instil 250 mL of 0.9% sodium chloride through a chest tube through a three‐way tap from a drip stand via gravity over 1 h without using an infusion pump.^3^
7. After 1 h, allow free flow of chest tube for 45 min.
8. Meanwhile, dilute 200 mg tyloxapol (4 ampoules = 20 mL) with 30 mL 0.9% saline and makeup to a total volume of 50 mL.
9. After 45 min post intrapleural saline irrigation, instil 50 mL (200 mg tyloxapol) diluted tyloxapol via chest tube over 5 min and clamp chest tube for another 45 min dwelling time.
10. Unclamp the chest tube after 45 min of intrapleural tyloxapol instillation and allow free flow of the chest tube until the next cycle.
11. The whole process (step 5 to step 10) can be repeated after 6–12 h from the first cycle based on clinical assessment of patient's condition and procedure outcomes.

In conclusion, the outcome of treatment for pleural infections can be enhanced with intrapleural interventions along with the standard best‐practice management. Treatment of pleural infection need to be individualized depending on availability of intrapleural medications, surgical risk, bleeding risk from intrapleural fibrinolysis, local expertise, financial status of patients and patient's preference. Further exploration and studies on the effectiveness and safety of intrapleural saline irrigation in combination with intrapleural tyloxapol are essential. Studies on a combination of fibrinolytic with tyloxapol (as an alternative to DNAse) are also lacking. The findings from our case series may serve as a basis for future larger prospective studies.

## AUTHOR CONTRIBUTIONS

Mas Fazlin Mohamad Jailaini and MFAH were involved with the concept, acquisition of data and drafting of manuscript. Mohd Jazman Che Rahim and Noor Amirah Saini were involved with literature review. Mohamed Faisal Abdul Hamid contributed to critical revision of important intellectual content. All authors had full access to data, contributed to the paper, approved the final revision for publication and take responsibility for its accuracy and integrity.

## FUNDING INFORMATION

The paper did not receive any specific grant from funding agencies in the public, commercial, or not‐for‐profit sectors.

## CONFLICT OF INTEREST STATEMENT

None declared.

## ETHICS STATEMENT

Written informed consent was obtained from the patient to publish this case report and the accompanying images.

## Data Availability

The data that support the findings of this study are available from the corresponding author upon reasonable request.
